# An Intelligent Non-Invasive Real-Time Human Activity Recognition System for Next-Generation Healthcare

**DOI:** 10.3390/s20092653

**Published:** 2020-05-06

**Authors:** William Taylor, Syed Aziz Shah, Kia Dashtipour, Adnan Zahid, Qammer H. Abbasi, Muhammad Ali Imran

**Affiliations:** 1 James Watt School of Engineering, University of Glasgow, Glasgow G12 8QQ, UK; S.Shah@mmu.ac.uk (S.A.S.); kia.dashtipour@glasgow.ac.uk (K.D.); a.zahid.1@research.gla.ac.uk (A.Z.); Qammer.Abbasi@glasgow.ac.uk (Q.H.A.); Muhammad.Imran@glasgow.ac.uk (M.A.I.); 2 Centre of Intelligent Healthcare, Coventry University, Coventry CV1 5RW, UK

**Keywords:** human motion detection, machine learning, random forest, KNN, SVM, neural networks, USRP, channel state information, real-time classification

## Abstract

Human motion detection is getting considerable attention in the field of Artificial Intelligence (AI) driven healthcare systems. Human motion can be used to provide remote healthcare solutions for vulnerable people by identifying particular movements such as falls, gait and breathing disorders. This can allow people to live more independent lifestyles and still have the safety of being monitored if more direct care is needed. At present wearable devices can provide real-time monitoring by deploying equipment on a person’s body. However, putting devices on a person’s body all the time makes it uncomfortable and the elderly tend to forget to wear them, in addition to the insecurity of being tracked all the time. This paper demonstrates how human motions can be detected in a quasi-real-time scenario using a non-invasive method. Patterns in the wireless signals present particular human body motions as each movement induces a unique change in the wireless medium. These changes can be used to identify particular body motions. This work produces a dataset that contains patterns of radio wave signals obtained using software-defined radios (SDRs) to establish if a subject is standing up or sitting down as a test case. The dataset was used to create a machine learning model, which was used in a developed application to provide a quasi-real-time classification of standing or sitting state. The machine-learning model was able to achieve 96.70% accuracy using the Random Forest algorithm using 10 fold cross-validation. A benchmark dataset of wearable devices was compared to the proposed dataset and results showed the proposed dataset to have similar accuracy of nearly 90%. The machine-learning models developed in this paper are tested for two activities but the developed system is designed and applicable for detecting and differentiating x number of activities.

## 1. Introduction

Human motion detection is an important area of research in the field of healthcare systems. Eventually, more and more sectors of the healthcare industry will begin to use technology [1,2]. In recent years, home healthcare through the use of different technologies has gained much attention for its ability to improve the lives of people who require special care [3,4]. Special care is required by a large number of people such as the elderly population. The elderly population is on the rise, leading to a substantial decline in nursing home capacity [5,6]. The elderly population is set to be 2.1 billion in the year 2050 according to statistics from the United Nations [7,8]. With this expected growth in the elderly population, it will have even more strain on the lack of caregivers, so that dependencies on the technology will be required to support the treatment [9]. The monitoring of elderly and vulnerable people can allow them to live more independently. This means that the level of care they receive can be less. This is because the monitoring can provide real-time messages to caregivers in the instance of a fall [10]. Human motion detection is the process of using technology to extract the features of the human movement [11,12,13]. Human motion detection can be used for the monitoring of patients and vulnerable people such as the elderly or young children [14,15]. Fall detection is just one example of how human motion can be used in the healthcare industry although an important example. The World Health Organization reports that falls can cause around 646,000 deaths and over 37 million serious injuries. [16,17]. If a system was able to provide careers with this information in real-time then the patient would be able to receive assistance from the carer without the carer having to be with the vulnerable person at all times contributing to a more independent lifestyle. Human movement can be detected by the use of wearable devices such as mobile or smartwatches using accelerometers, which can then pass the information to carers or physicians etc. [18,19]. This leaves an issue of when the patient forgets to wear the wearable device. Another method of human motion detection is to use radio waves already in the atmosphere such as Wi-Fi in a home network. This technique is considered as non-invasive. Non-invasive is defined in medical terms as not involving the introduction of instruments into the body such as the case with wearable devices. This can be achieved by using the Channel State Information (CSI) from Wi-Fi to look at the amplitude of the CSI as a human moves between the radio waves [20,21]. The CSI is a feature in Wi-Fi that describes how the wireless signal propagates between the transmitting node and receiving node [22]. This data can be exploited to detected changes during a specific human motion. This research will explore the use of Universal Software-defined Radio Peripheral (USRP) to build a dataset of the CSI information of human activities and then use machine learning for binary classification of a human either sitting down or standing up. USRPs will be used because they offer a simple framework for experimentation rather than setting up complex systems for functionality testing [15,23]. USRPs are widely used in research applications because of their ability to transfer and receive frequencies in several bands [24]. URSPs provide flexibility as they can be tuned to a wide range of frequencies [25]. This work will use 64 subcarriers. Orthogonal frequency division multiplexing (OFDM) is used for 64 points of fast Fourier transformer (FFT) producing 64 frequency carriers (subcarriers) [26]. Lower frequencies are able to detect the smaller movements while higher frequencies are able to detect larger movements [27]. Using USRPs allows for a range of frequencies to be used in the experimentation which will allow a greater detection in movements overall. This paper aims to research the abilities to use RF signals to be able to classify human motion in a real-time application. This paper reports two major contributions to the state of the art. The first contribution is presenting a simple set up of how a machine learning model can provide real-time classification on human motion using data retrieved from a URSP. The second contribution is providing a comparison between the newly acquired dataset and an existing wearable device human motion dataset. This paper is organized in the following sections. Section 2 will detail some of the related work. Section 3 will detail the methods employed to collect the data. Section 4 will describe the methods of machine learning used and Section 5 will display the results and discuss said results as well as compare the results to a benchmark dataset collected from wearable devices.

## 2. Related Work

This section looks at the recent literature in various forms of human motion detection and where machine learning has been applied. The articles in [28,29,30] collected a range of human activities where the test subjects were using wearable accelerometer on their wrists. The dataset collected by these activities was then run through the machine learning algorithms of Random Forest, K Nearest Neighbours (KNN) and Support Vector Machines (SVM). The results found that the Support Vector Machine had the highest results of 91.5%. The work of [31,32,33] used frequency-modulated continuous-wave (FMCW) radar system to look at the Doppler, temporal changes and radar cross-sections to collect data of falling and other fall-related activities such as stepping, jumping, squatting, walking and jogging from three participants. The data was then run through 10 cross-fold validation with KNN to achieve a high accuracy result of 95.5%. This work demonstrates that wireless waves can be used to classify human motion through the changes in frequencies. A similar work was done on multi-channel extraction in [34,35]. Jalal et al. [36] used a benchmark dataset of 14 indoor human activities. The benchmark dataset was collected using triaxial accelerometer sensors. The research included separating the static activities from the dynamic activities. The paper then went on to apply the random forest algorithm for machine learning classification. The static results scored higher at 92.16% with the dynamic activities scoring 80.0% with an average result of 85.17%. The work conducted in [37] used wearable smartwatches to monitor the movement of ping-pong players. The watch recorded data of eight different motions on how the test subjects moved the ping-pong paddle including forehand attack, forehand flick, backhand flick etc. The data was then processed using seven machine learning algorithms including Random Forest, SVM, KNN and decision trees. The research found the Random Forest to be the best performance with an accuracy score of 97.80%. The paper [38] made use of CSI on Wi-Fi OFDM signals for the classification of five different arm movements. The human-made different arm movements while standing between a Wi-Fi router and a laptop sending wireless signals to each other. The CSI was then captured and machine learning was applied to the collected data. The machine learning algorithm chosen was the Long Short-Term Memory (LSTM) which was able to achieve a high-accuracy result of 96%. A similar work on healthcare was done in [39,40,41]. Nipu et al. [42] used CSI information to try and identify a specific person. The experiment conducted had different people walk through two devices while data is transmitted and store the CSI information obtained while that person walked through the radio frequencies. The dataset was then passed through the machine learning algorithms, Random forest and Decision tree. The experiments found that the algorithms scored higher when only two people were used in a binary classification experiment.

## 3. Collection of Data

**Figure 1:**
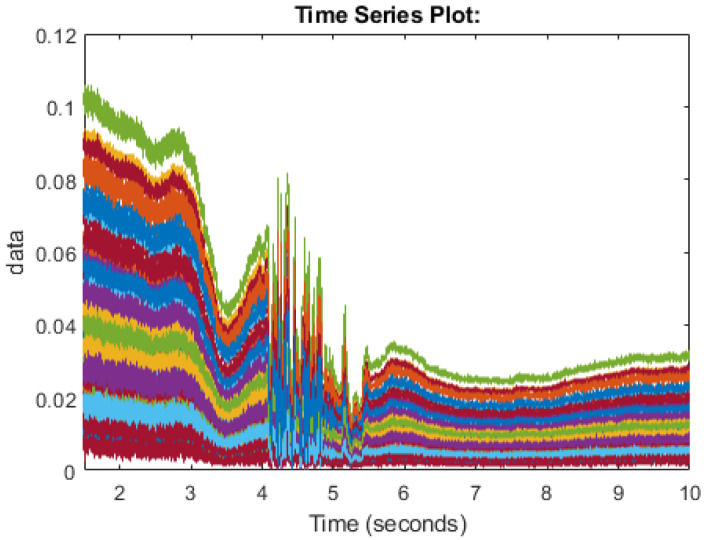
Channel State Information for the human motion of sitting down.

**Figure 2:**
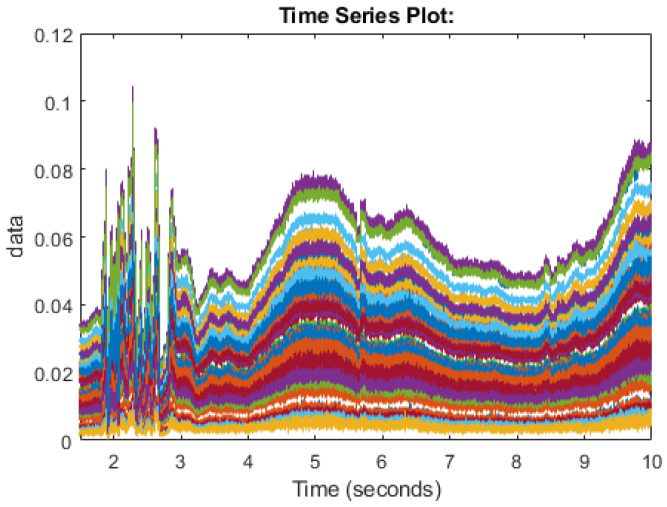
Channel State Information for the human motion of standing up.

In this section, we will discuss the methods of how the data is collected. The work of this paper makes use of Universal Software Radio Peripheral (USRP) devices to send packets between antennas [43]. Two USRPs were used, namely the X310/X300 models from a national instrument (NI), each equipped with extended bandwidth daughterboard slots covering DC– 6 GHz with up to 120 MHz of baseband bandwidth. The X300 model was used as the transmitter with the X310 model performing as the receiver. The devices were connected to two PCs through 1G Ethernet cable connections. The USRP’s were equipped with two VERT2450 omni-directional antennas. The simulation was designed using MATLAB/Simulink program linked to the USRP’s. The experiment was undertaken in an office environment and USRPs were kept at 4 m within line of sight with each other, to achieve the best performance. Experiments were performed with set parameters. Table 1 lists the parameters of the software configuration of the USPRs. The USRPs used in the study have a frequency range from 1 GHz to 10 GHz. Center frequency for the USRPs was set as 5.32 GHz and the operational frequency of omni-directional antenna was also 5.32 GHz, with 3 dBi gain. The gain of USRP chosen to be 70 for transmitter and 50 for the receiver. The hardware parameters values of the USRP is summarised in Table 2. Ethical approvals of participants have been acquired through the University of Glasgow ethic review committee. The participants were asked to perform the different human motions in this research of standing up and sitting down. Participants completed the task multiple times to be able to collect many samples of the CSI information to allow for error and allow cleanest samples to be taken forward. The test was performed in an 7-by-8 m office space containing furniture such as tables, chairs, draws, etc. The human motion is then carried out between the antennas and the Channel State Information is then recorded while this human motion is carried out. As radio signal propagation is proportionate to the movement of the human, the CSI will differentiate as different motion takes place. The CSI will show certain properties when a certain movement is made by the human. In this paper, we have recorded the CSI for multiple subjects sitting down on a chair and then standing from a chair. As there are many variations in the way the signals propagate and human movement will never be exactly the same, the movement should follow the same patterns in the CSI data. Some samples can be considered as good samples where interference is set to a minimum and some samples may be affected by ambient movement or atmosphere factors. Multiple samples are taken to try to capture the flow of the patterns and machine learning is used to attempt to classify the samples. The final dataset contains 30 samples each of sitting and standing. Figure 1 and Figure 2 display the CSI of the 64 subcarriers of the USRP. Each color represents a subcarrier and the frequency of the subcarrier is shown along the Y-axis and time is shown along the X-axis while an activity is taking place. Figure 1 shows the pattern followed in a good sample of sitting down and Figure 2 shows the pattern followed in a good sample of standing up.

**Figure 3:**
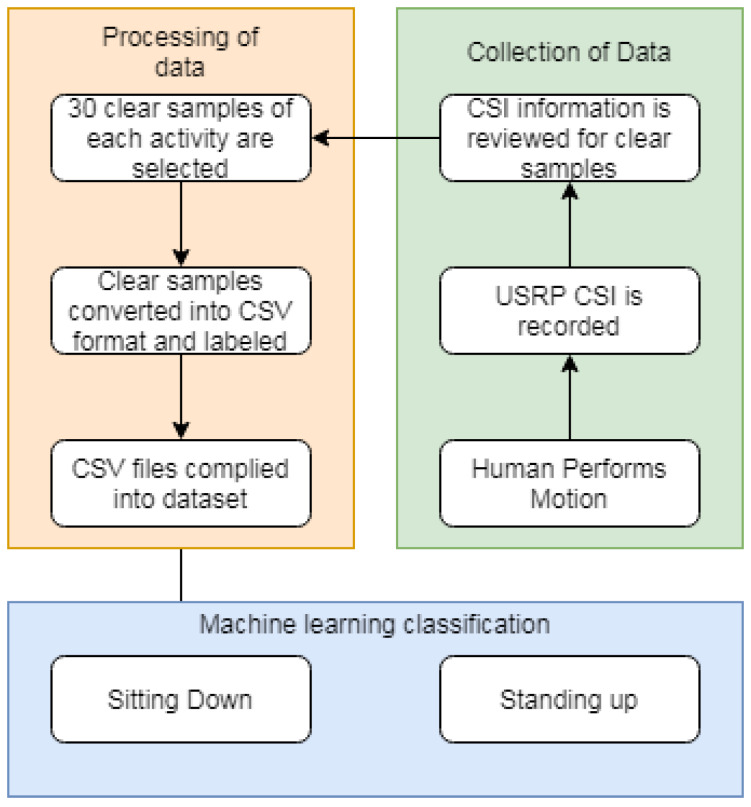
Experiment flow chart.

The USRPs are configured to transmit data from one antenna to the other for 10 s. As the signals propagate in different ways each time a sample is taken, the number of packets received has slight variations. However, this has little effect as the aim is to detect patterns in the radio waves as a certain human motion is carried out during the transmission of packets. Figure 3 details the process used in this experimentation.

## 4. Machine Learning Process

The dataset performance has been measured using a range of machine learning algorithms using the Python SciKit library. Scikit is a machine learning package that is widely used in the data science field [44]. The Samples are converted into CSV format so that they can be processed using the SciKit library. The Python library Pandas is used to process the CSV files. Pandas imports the CSV files as dataframes within Python which the SciKit library then processes [45]. The labels are added as the first column on the dataframes as the data is of varying length throughout the samples. Then the dataframe of each sample is combined together to make the full dataset, the varying lengths result in NAN values being part of the dataset. To resolve this issue SciKit provides a function called simple imputer. This is used to replace all NAN values with a 0. Therefore the shorter samples of the dataset will contain 0 values tailing the row on the CSV file. This is not perceived to be a problem as the different lengths are minor and the pattern of the RF signals is still apparent. This is part of the variance between different samples. The data set is then divided into two variables, one for the labels and one for the data itself. Then the four machine learning algorithms are declared. The four algorithms used to test this dataset are Random Forest, K nearest Neighbours, Support Vector Machine and Neural Networks. The ensemble classifier takes each algorithm prediction as a vote and then whichever prediction has the most votes will be the prediction declared by the ensemble classifier.

Random forest is a collection of decision trees. Each tree makes a prediction of the output by taking in looking for features found in the training phase. This prediction is considered a vote. The majority of predictions is the final Random Forest prediction [46]. Equation (1) shows how SciKit uses Random Forest:

(1) $$Ni_{j}=W_{j}C_{j}-W_{left(j)}C_{left(j)}-W_{right(j)}C_{right(j)}$$

- $Ni_{j}$ = the importance of node *j*
- $W_{j}$ = weighted number of samples reaching node *j*
- $C_{j}$ = the impurity value of node *j*
- $left_{j}$ = child node from left split on node *j*
- $right_{j}$ = child node from right split on node *j*

The K nearest Neighbours algorithm is known for its simplicity. The algorithm works by comparing the testing data to the training data [47]. The features of the training data are assigned a K sample then the testing data is assigned to the K sample that most closely matches the new data [48]. Equation (2) shows the Euclidean KNN equation which is the default method for SciKit:

(2) $$\sqrt{\sum_{i=1}^{k}{\left(x_{i}-y_{i}\right)}^{2}}.$$

- *k* = is the number of samples
- *x* = the data
- *y* = the label

The Support Vector Machine algorithm works by constructing hyper planes and uses these hyper planes to separate the input data into different categories. The training data is used to train the hyper planes based on features of the training data [49]. Equations (3) and (4) shows how SVM works:

(3) positiveequation=w.u+b>0

(4) negativeequation=w.u+b<0.

- *w* = the vector per perpendicular to median of hyper-plane
- *u* = the unknown vectors
- *b* = b is constraint

The Neural Network model is inspired by the human brain [50]. A neural network consists of an input layer, hidden layer and output layer which are all interconnected. The aim is to transform a set of inputs to the desired outputs by using weights associated with the neurons in the hidden layer [51]. When the neural network passes the training input, the output is observed. If the output is incorrect then the hidden layer is adjusted until the correct output is achieved. Then the testing data can be passed through the model as the input data and the output is the prediction [52].

(5) $$f\left(b+\sum_{i=1}^{n}x_{i}w_{i}\right).$$

- *b* = bias
- *x* = input to neuron
- *w* = weights
- *n* = the number of inputs from the incoming layer
- *i* = a counter from 0 to n

Two experiments are done using each algorithm on the dataset. The first experiment makes use of 10 fold cross-validation. The 10 fold cross-validation is used to test machine learning models where the data is divided into training and testing data. The number 10 refers to the number of groups. Each group takes a turn as the test data and the rest of the groups are used as training data. This ensures that there is variance in the test data. The results of the 10 runs are then averaged to give the final results [53]. The second experiment uses the train test split method where the dataset is split 70/30. We used 70% of the dataset to train the dataset and 30% of the dataset is used for testing. The results of this paper will use the performance metrics of Accuracy, Precision, Recall and F1-score. These performance metrics are calculated by looking at four classification values. The classification values are True Positive (TP), True Negative (TN), False Positive (FP) and False Negative. The equations for how the performance metrics are calculated are shown in Equations (6)–(9).

The accuracy displays the total number of correct classifications versus the total classifications made.

(6) $$Accuracy=\frac{TP+TN}{TP+TN+FP+FN}.$$

Precision metric is used to measure one of the classifications against how precise it is in comparison to all classifications. The results are presented as an average between both sitting and standing.

(7) $$Precision=\frac{TP}{TP+FP}.$$

The recall wasused to show the ratio of the correct classification to all classifications for that label. This was run for both sitting and standing and presented as an average.

(8) $$Recall=\frac{TP}{TP+FN}.$$

The F1-score is used to provide an average between the Precision and Recall Metrics.

(9) $$F1-score=2X\frac{Precision*Recall}{Precision+Recall}.$$

## 5. Results and Discussion

This section presents the output of the machine learning algorithms after they have completed 10-fold cross-validation and train test split using the Python variables containing the data and comparing the prediction of the data to the actual labels of the data. The performance metrics used to compare the algorithms include the accuracy score as well as precision, recall, and f1 score. A confusion matrix is also provided to show how each sample has been classified.

### 5.1. Cross-Validation

In Table 3 it can be seen that the best accuracy is from Random Forest followed by the neural network. Although both KNN and Support Vector Machine still has high accuracy. When the algorithms were compiled together in the ensemble classifier, the accuracy was 92.18%. The accuracy was calculated as an average of the 10 sets of testing data used in each of the 10 cross-fold validation process. The dataset is made up of 30 samples each of sitting and standing which each contain 64 subcarriers. So the total number of rows contained in the dataset is 3840 subcarriers. The confusion matrix is a table used to describe how an algorithm has performed. The confusion matrix shows exactly how many samples were classified in which category. The Y axis on the confusion matrix represents the prediction of the algorithm and the X axis represents the actual classification.

**Figure 4:**
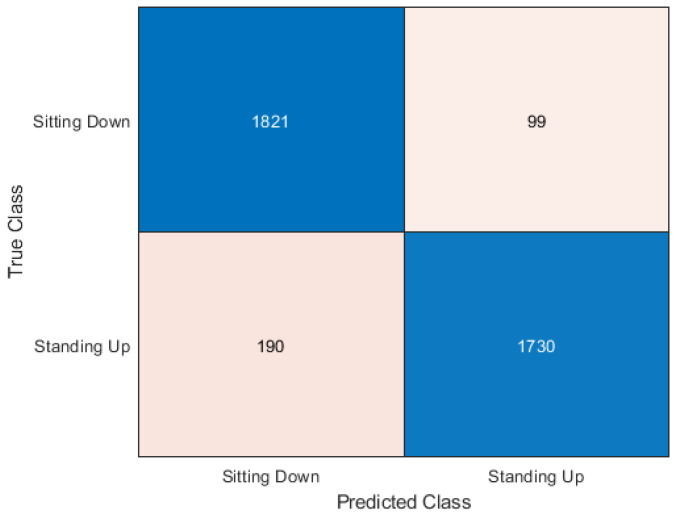
Confusion matrix for random forest.

The Random Forest algorithm was the best performer out of all the algorithms. It can be seen in Figure 4 how the 3840 samples have been classified. We classified 1821 sitting samples as sitting. This is represented in the top left square where the X-axis matches the Y-axis. Then, 99 sitting samples were incorrectly classified as standing. This is where the X-axis and Y-axis mismatch. The majority of sitting samples were correctly classified so this shows good results. The classification of standing samples was slightly less accurate but still good results. 190 samples were classified incorrectly as sitting, which is higher than the 99 sitting samples incorrectly classified as standing. This leaves the remaining 1730 standing samples as being correctly classified.

**Figure 5:**
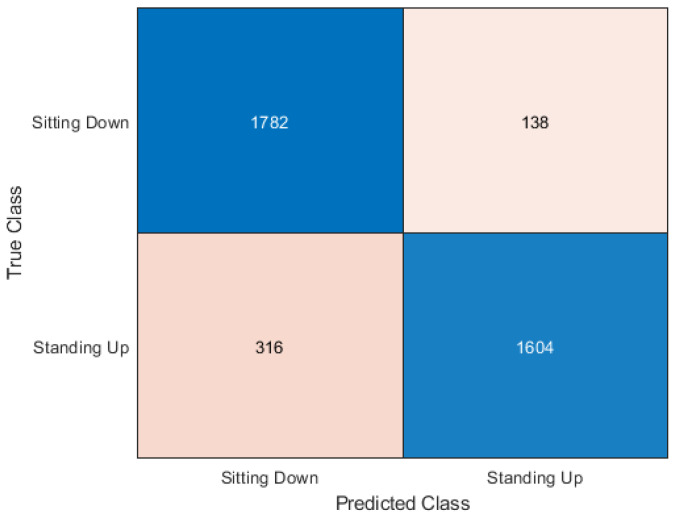
Confusion matrix for K Nearest Neighbours (KNN).

The KNN algorithm had an accuracy score of 88.17%, which is only around 4% less than Random Forest. In the confusion matrix shown in Figure 5 it can be observed on how much the classifications differ in the 4% difference in accuracy. It appears that both algorithms had better classification results with sitting over standing. KNN had 138 sitting subcarriers incorrectly classified as standing but had 316 standing classifiers incorrectly classified as sitting. However, the majority of subcarriers were classified correctly.

**Figure 6:**
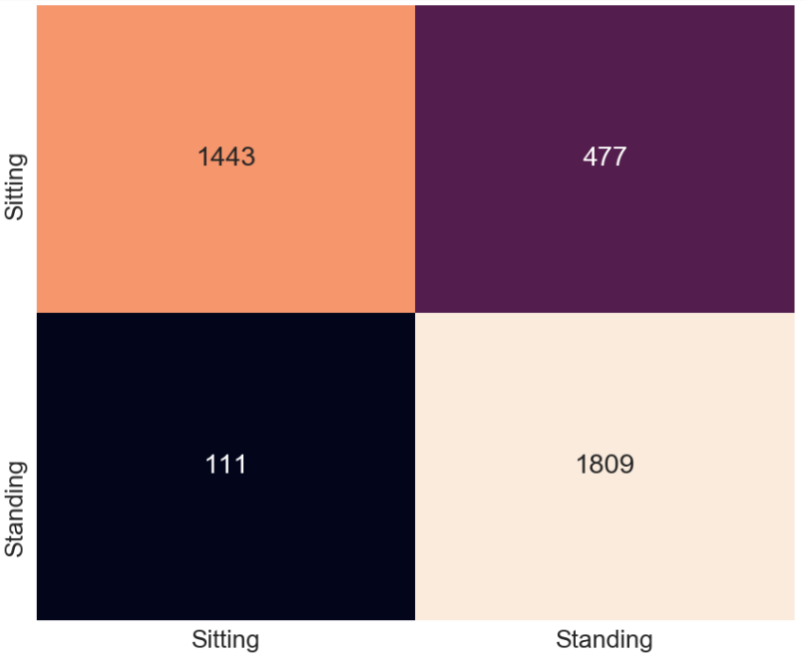
Confusion matrix for Support Vector Machines (SVM).

Support Vector Machine was the lowest scoring algorithm in this experiment but with an accuracy score of 84.68%, the majority of samples were classified correctly. Unlike Random forest and KNN, SVM showed better performance with the standing up samples. Only 111 of the standing subcarriers were wrongly classified as sitting down. We classified 477 sitting-down samples incorrectly as standing, as shown in Figure 6.

**Figure 7:**
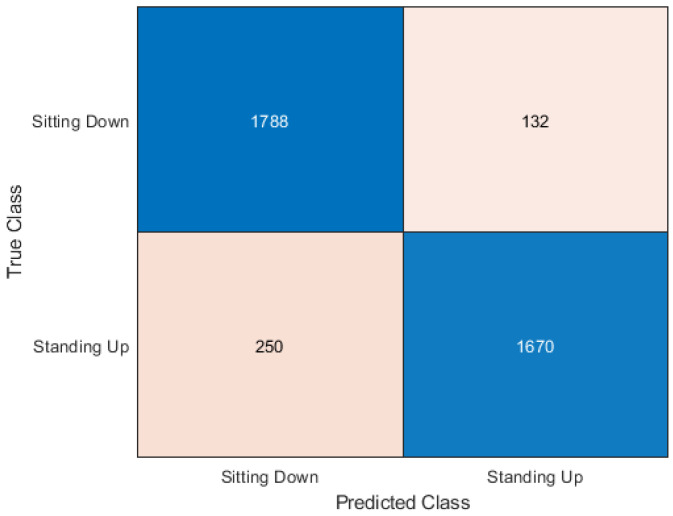
Confusion matrix for neural networks.

The Neural Network classifier had the second-best accuracy score of 90.05%. Like Random forest and KNN, it had better performance with sitting down samples. The confusion matrix shown in Figure 7 shows only 132 sitting samples were incorrectly classified compared to the 250 standing samples classified incorrectly.

**Figure 8:**
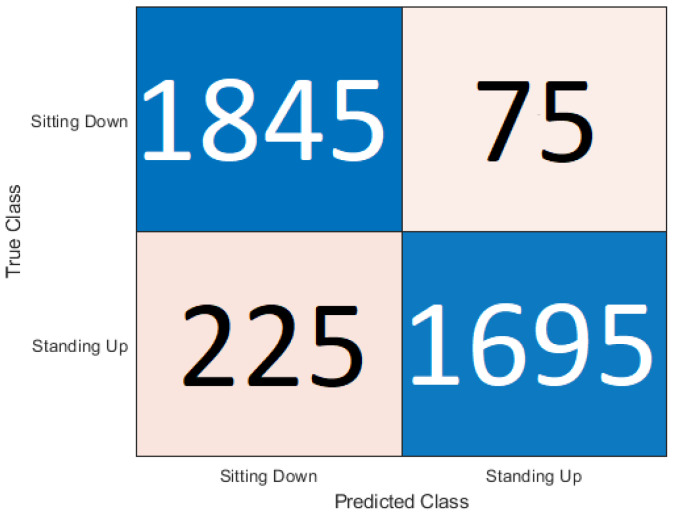
Confusion matrix for ensemble classification.

The confusion matrix for the ensemble classification is shown in Figure 8. The ensemble had the best performance with the sitting down samples with only 75 of the samples being classified as incorrect. The ensemble classifier was let down by the standing up samples as it incorrectly classified 225 samples. It can be seen that the ensemble technique worked well with the sitting down samples but was not so good with the standing up samples. Support Vector Machine had the lowest error rate for standing up samples.

### 5.2. Train Test Split

In Table 4 it can be seen that the best accuracy is still Random Forest followed by the neural network. Although both KNN and Support Vector Machine still have high accuracy, when the algorithms are compiled together in the ensemble classifier the accuracy is 93.83%. The accuracy is calculated by comparing the 30% test data predictions to the actual labels of the data. The full dataset is made up of 30 samples each of sitting and standing which each contain 64 subcarriers. So the total number of rows contained in the dataset is 3840 subcarriers. 1152 subcarriers is the number of the 30% test samples used in the train test split method rather than the whole dataset being used testing data at some point. In the testing data there are 512 standing up samples and 640 sitting down samples. The confusion matrix in this experiment shows only the 1152 samples, the total number of tested samples.

**Figure 9:**
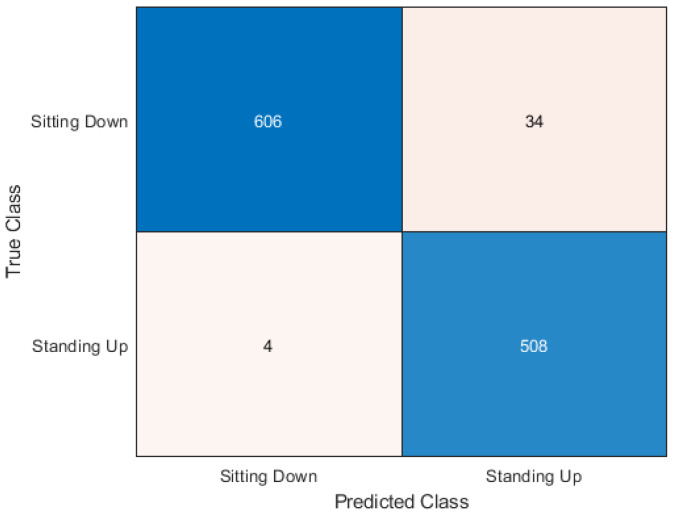
Confusion matrix for random forest.

The Random Forest algorithm was the best performer out of all the algorithms. It can be seen in Figure 9 how the 1152 samples have been classified. 606 sitting samples were correctly classified as sitting. This is represented in the top left square where the X-axis matches the Y-axis. Then 34 sitting samples were incorrectly classified as standing. This is where the X-axis and Y-axis mismatch. The majority of sitting samples were correctly classified which is a positive result. The classification of standing samples was more accurate than sitting in contrast to the cross-validation results. Only four samples were classified incorrectly as sitting, which leaves the remaining 508 standing samples as correctly classified.

**Figure 10:**
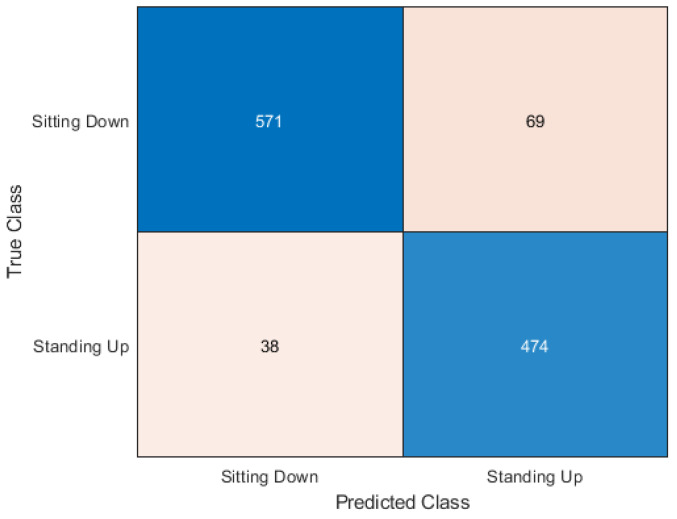
Confusion matrix for KNN.

The KNN algorithm had an accuracy score of 90.71%, which is an improvement over the cross-validation experiment. In the confusion matrix shown in Figure 10, KNN, just like Random Forest, performed better with the standing-up samples rather than the sitting-down samples. KNN had 69 sitting subcarriers incorrectly classified as standing, but had only 38 standing classifiers incorrectly classified as sitting. However, the majority of subcarriers were classified correctly.

**Figure 11:**
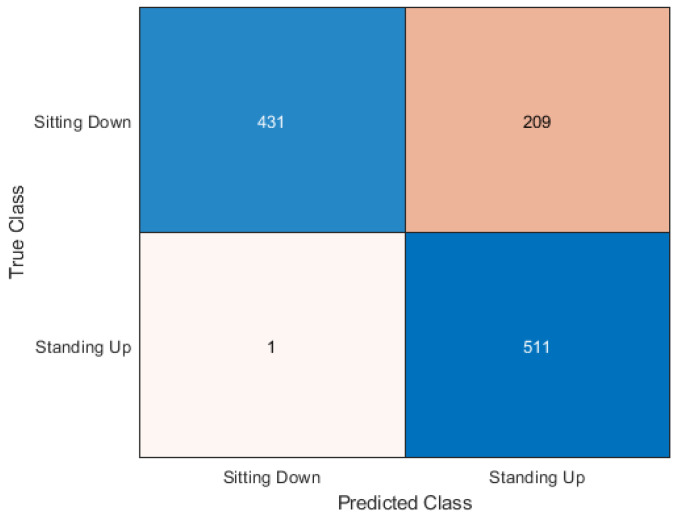
Confusion matrix for SVM.

Support Vector Machine was the lowest scoring algorithm in this experiment, but with an accuracy score of 81.77%, the majority of samples were classified correctly. Like Random forest and KNN, SVM showed better performance with the standing up samples. Only one of the standing subcarriers was wrongly classified as sitting down however 209 sitting down samples were classified incorrectly as standing, as shown in Figure 11.

**Figure 12:**
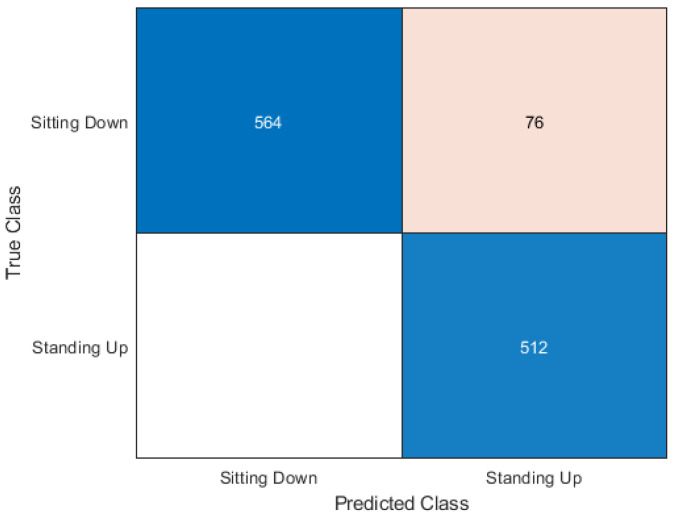
Confusion matrix for neural networks.

The Neural Network classifier had the second-best accuracy score of 93.40%. Like the other algorithms, it had better performance with standing-up samples. The confusion matrix shown in Figure 12 shows 76 sitting samples were incorrectly classified compared to the 0 standing samples classified incorrectly.

**Figure 13:**
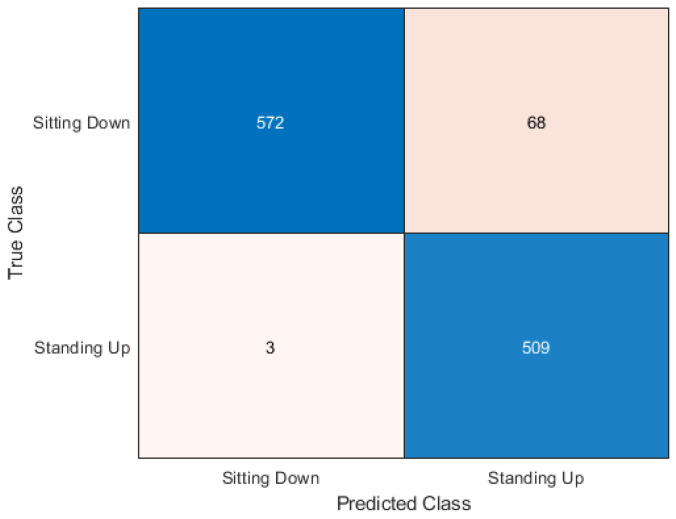
Confusion matrix for ensemble classification.

The confusion matrix for the ensemble classification is shown in Figure 13. The ensemble method shows better performance with the standing samples as expected as all the algorithms performed better with the standing samples. The ensemble method gave a good average number for the incorrect sitting samples preventing it from going too high, making use of the voting system.

### 5.3. Comparison of Cross-Validation and Train Test Split

**Figure 14:**
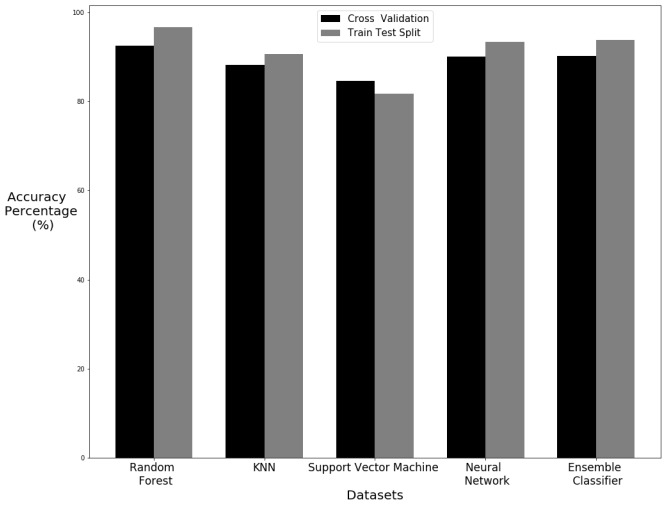
Comparison of cross validation and train–test split.

The difference in accuracy can be seen in Figure 14. The train test split shows better classification results with the standing up samples. This is because there are more standing up samples within the 70% training set. This shows that the more training on a sample gives better results. All of the algorithms have higher accuracy except from SVM with the train test split. Cross-validation, however, gives a better representation of the algorithm performance since all of the data takes a turn for training and testing so every possible combination is tested.

### 5.4. Real Time Classification

For Real-Time classification of data, the dataset needs to be used to create a model. Random forest provided the highest accuracy results, it was chosen to create the model. Instead of dividing the dataset into 10 groups for cross-fold validation, the whole dataset was used for training. This allows for the model to have the most amount of training. The SciKit Python package allows for models to be saved and recalled later by using the Joblib package. Flask was used to create a web interface that could action Python scripts.

**Figure 15:**
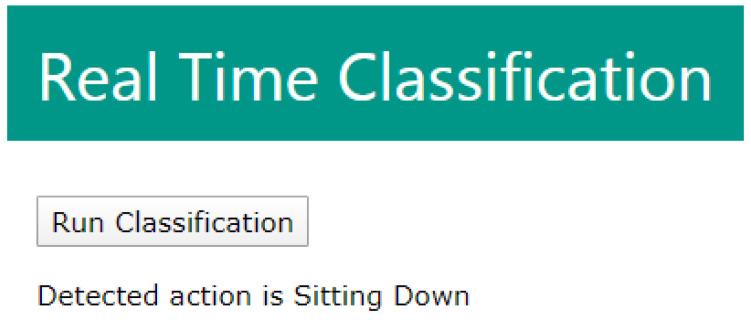
Flask web interface displaying classification result.

**Figure 16:**
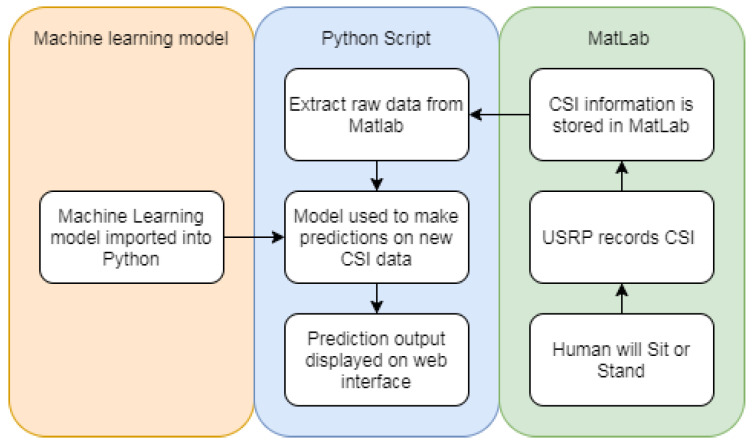
Flask web interface process.

The application works when the user presses the “Run Classification” button. The button then actions a Python script within the Flask app. The Python script works by connecting to the Matlab session that records the CSI from the USRP. The Matlab session will need to be shared and then Python can connect and access the variables stored on Matlab. When an experiment is run on the USRP the CSI is stored in a timeseries called CSI in Matlab. The Python script first activates a Matlab script which then extracts the raw CSI data from the timeseries. Once the raw data is stored on a variable in Matlab the Python script can access the variable and apply the previously saved model to make classifications on the new data obtained from the USRPs. As this process takes place the interface will display “Loading...” as the output. To test the real-time application additional samples of sitting down and standing up were taken. Six of each group were taken to give a total of 12 samples. These 12 samples were completely unseen when training the model as they were not contained in the dataset. The trained model was able to correctly classify all of these samples. As seen in Figure 15, the classification is displayed as the output after the script has run. This web application has proved to be able to access the Matlab variable that contains the CSI obtained from the USRP and make classifications using a previously stored model. The real-time web application is able to be extended to make any amount of classifications as it is based on the model used to make the classifications of newly received data. Figure 16 details the process undertaken by the real-time application web interface.

### 5.5. Benchmark Dataset

**Figure 17:**
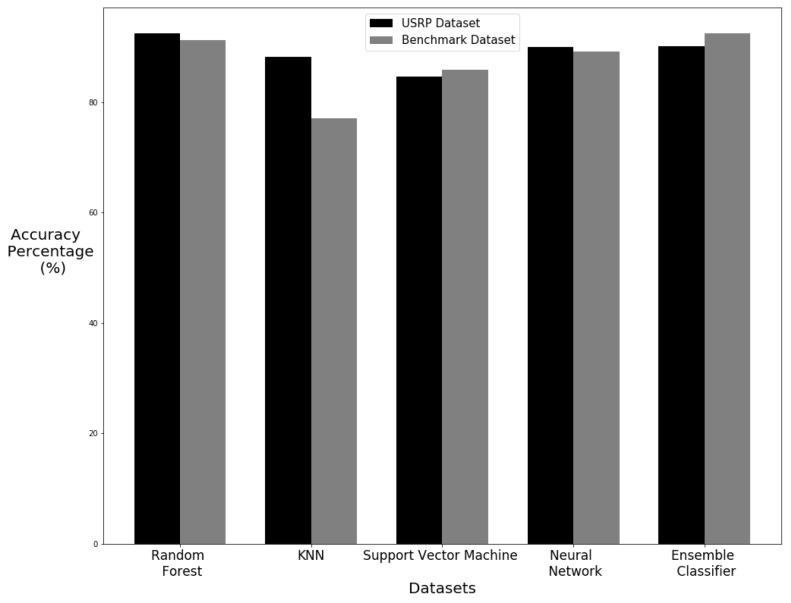
Comparison of results with cross validation.

**Figure 18:**
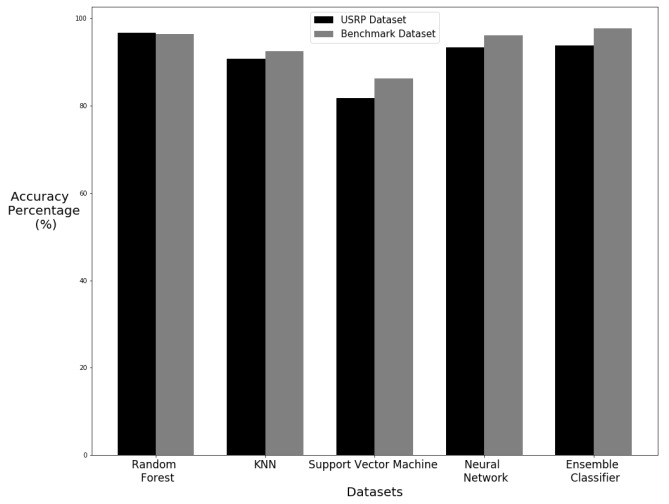
Comparison of results with train test split.

As the machine learning results for the dataset were of high accuracy, it evidences that CSI is a viable method for human motion detection. For a comparison of how effective CSI can be used to identify human motion, we have compared the machine learning results of this dataset to that of another dataset. Ref. [54] have published a dataset detecting a range of human motions using smartphones which are equipped with accelerometers. The machine learning process used with the USRP dataset created in this research has been applied to this benchmark training dataset. This comparison gives a good indication of how non-interference detection compares to wearable devices in the field of human motion detection. The results show that the USRP dataset is able to provide similar results to the benchmark dataset which is using wearable devices. The Random Forest algorithm displays similar results. The accuracy values are shown in Table 5 and Table 6 for cross-validation and train test split experiments respectfully. Figure 17 and Figure 18 give a visual representation of the differences between the two datasets for cross-validation and train test split experiments respectfully. The Random Forest was the best performer in both sets of data with both cross-validation and train test split methods. KNN performed much better using the USRP dataset with a cross-validation but was lower with the train–test split. Support Vector Machine had similar performance within the two datasets with only a larger difference in accuracy between datasets using the train split method. The Neural Network algorithm also had a small difference between datasets with a slight increase with the cross-validated USRP dataset but a larger difference in favor of the benchmark dataset when using the train test split. The ensemble classifier actually performed better with the benchmark dataset in both methods but by only a small difference when using the cross-validation method. Such findings demonstrate that the USRP is capable of producing similar results and even higher precision scores compared to a dataset obtained using wearable devices. The primary reason that the datasets collected using USRP outperforms the wearable devices datasets is that USRP leverage on multiple frequency subcarriers. An intricate change in wireless medium is picked up by the multiple carrier USRP model, whereas the wearable devices such as accelerometer and magnetometer are not sensitive enough to detect body motion. That is why, due to high sensitivity against body motion, the USRP works better in detecting body movements.

## 6. Conclusions

In this paper, we have proposed an algorithm and dataset which can be used in the detection of human motion. The dataset includes observations of the channel state information from USRPs as human activities take place between the antennas. The dataset is designed for binary classification between sitting down and standing up human motion. The performance of machine learning shows good results with the Random Forest algorithm producing a high accuracy result of 92.47%. The high-accuracy in the results show that there is a significant difference between the CSI information of standing up and sitting down for a machine algorithm to be able to establish the difference. The web application was able to successfully classify samples of data that were absent during the learning phase directly from the Matlab session which contained the CSI directly from the USRP. The use of USRP data to detect human motion was also compared to a benchmark dataset where human motion was detected using wearable devices. The same machine-learning techniques were applied to the benchmark dataset and the results show good accuracy with the benchmark dataset.

## Figures and Tables

**Table 1:** Software configuration parameters selection.

Parameters	Values
Input data (Signal)	round(0.75*rand(104,1))
Sample time	1/80e4
Modulation type	QPSK
Bit per symbol M	2 bits
OFDM Subcarrier	64 subcarriers
Pilot subcarrier	4
Null subcarrier	12
Cycle prefix M	NFFT-data subcarrier
Samples per frame	Used subcarrier log2 (M)

**Table 2:** Hardware configuration parameters selection.

Parameters	Values
Platform	USRP X300/X310
TX IP address	192.168.11.1
RX IP address	192.168.10.1
Channel mapping	1 TX, 2 RX
Centre frequency	5.32 GHz
Local oscillator offset	Dialog
PPS source	Internal
Clock source	Internal
Master clock rate	120 MHz
Transport data type	Int16
Gain (dB)	TX 70, RX 50
Sample time	1/80e4
Interpolation factor	500
Decimation factor	500

**Table 3:** Cross-validation results.

Algorithm	Accuracy	Precision	Recall	f1-Score
Random Forest	92.47%	0.93	0.92	0.92
K nearest Neighbours	88.17%	0.89	0.88	0.88
Support Vector Machine	84.68%	0.86	0.85	0.85
Neural network model	90.05%	0.90	0.90	0.90
Ensemble Classifier	92.18%	0.92	0.92	0.92

**Table 4:** Train test split results.

Algorithm	Accuracy	Precision	Recall	f1-Score
Random Forest	96.70%	0.97	0.97	0.972
K nearest Neighbours	90.71%	0.91	0.91	0.91
Support Vector Machine	81.77%	0.87	0.82	0.82
Neural network model	93.40%	0.94	0.93	0.93
Ensemble Classifier	93.83%	0.94	0.94	0.94

**Table 5:** Comparison of results with cross validation.

Algorithm	USRP Dataset Accuracy	Benchmark Dataset Accuracy
Random Forest	92.47%	91.20%
K nearest Neighbours	88.17%	77.06%
Support Vector Machine	84.68%	85.90%
Neural network model	90.05%	89.21%
Ensemble Classifier	92.18%	92.40%

**Table 6:** Comparison of results with train test split.

Algorithm	USRP Dataset Accuracy	Benchmark Dataset Accuracy
Random Forest	96.70%	96.49%
K nearest Neighbours	90.71%	92.48%
Support Vector Machine	81.77%	86.21%
Neural network model	93.40%	96.11%
Ensemble Classifier	93.83%	97.74%
